# Exploring Logical Intuition in Base-Rate Problems Using the Instructional Manipulation Paradigm

**DOI:** 10.3390/bs15010083

**Published:** 2025-01-18

**Authors:** Debiao Zhu, Ping Lu, Zhujing Hu, Jianyong Yang, Dandan Nie

**Affiliations:** 1 School of Psychology, Jiangxi Normal University, Nanchang 330022, China; huzjing@jxnu.edu.cn (Z.H.); niedandan@jxnu.edu.cn (D.N.); 2 School of Education, Jiangxi University of Technology, Nanchang 330022, China; luping@jxut.edu.cn (P.L.); yangjianyong@huanghuai.edu.cn (J.Y.)

**Keywords:** dual-process theories, logical intuition, base-rate problem, instructional manipulation

## Abstract

The default–interventionist model of dual-process theories proposes that stereotype descriptions in base-rate problems are processed using Type 1 processing, while the evaluation of base rates depends on Type 2 processing. The logical intuition view posits that people can process base-rate information using Type 1 processing. This study examined the logical intuition view using the instructional manipulation paradigm. Participants judged the probability that a character in a base-rate problem belonged to a particular group based on either their beliefs or statistics and then rated their confidence in their responses. Results showed that a belief–statistics conflict affected both statistics- and belief-based judgments, resulting in lower probability estimates, longer response times, and lower confidence ratings for conflict items compared to no-conflict items, suggesting participants intuitively processed base rates such that they influenced rapid belief judgments. This intuitive logic effect was observed for extreme base rates, moderate base rates, and moderate base rates with small absolute values. These findings are inconsistent with the default–interventionist model but align with dual-process theories emphasizing logical intuition. The study provides additional evidence for human rationality.

## 1. Introduction

People often disregard rationality when reasoning and making decisions, leading to biased responses that violate simple rules of logic (Kahneman, 2011). Take the following base-rate problem as an example: Jo is a randomly selected individual from a study with 995 nurses and five doctors. Jo, aged 34, resides in a luxury villa, is eloquent, interested in politics, and highly focused on career development. Is it more probable that Jo will be a doctor or a nurse? (Kahneman & Tversky, 1973). Extensive studies have shown that people tend to answer that Jo is a doctor, apparently relying on stereotypes and ignoring the extremely low rate of doctors compared to nurses in the description (Evans & Stanovich, 2013; Tversky & Kahneman, 1974).

The default–interventionist model of dual-process theories offers an appealing explanation for base-rate neglect, suggesting that reasoning and decision-making are influenced by two types of processing, termed Type 1 and Type 2. Type 1 processing is intuitive, fast, independent of working memory, and deals with superficial cues (e.g., stereotypes in base-rate problems), while Type 2 processing is analytic, slow, relies on working memory, and applies logical rules (e.g., considering base rates in base-rate problems) (Evans, 2008; Evans & Stanovich, 2013; Singmann et al., 2016). Base-rate neglect occurs because individuals tend to conserve working memory, which reduces engagement of Type 2 processing and increases reliance on Type 1 processing, leading to a higher likelihood of producing responses based on stereotypes (Kahneman & Frederick, 2002; Kahneman & Tversky, 1973).

However, several authors have disputed the assumption that there is a direct match between the type of cognitive processing used and the response given, proposing that individuals can process logic rules based on intuitive Type 1 processing (De Neys, 2014; Howarth et al., 2022; Trippas et al., 2016). This form of processing is known as logical intuition.

Evidence for logical intuition in base-rate problems comes primarily from conflict detection studies. These studies involve participants responding to problems with and without conflict. In conflict problems, heuristic intuition elicits responses that conflict with logical thinking. In no-conflict problems, heuristic intuition and logical consideration suggest the same response (see Table 1). When responding to conflict problems, as compared to no-conflict problems, participants show longer response latencies (Frey et al., 2018), lower confidence in their estimates (De Neys et al., 2011), more repeated examination of critical problem elements (De Neys & Glumicic, 2008), and greater engagement of the anterior cingulate cortex, linked to error and conflict detection sensitivity (De Neys et al., 2008). These effects occur regardless of whether answers follow logical rules or not (De Neys et al., 2011; Zhu et al., 2023). Conflict detection effects persist even in situations where analytic Type 2 processing is minimized by time pressure or cognitive load (Bago & De Neys, 2017; De Neys, 2014). If participants did not process logical rules, there would be no difference in responses between conflict and no-conflict problems. Thus, a plausible explanation for the above phenomenon is that participants can intuitively process logical rules.

Handley et al. (2011) have argued, however, that the effects of intuitive logic obtained from conflict detection studies may have stemmed from participants being asked to respond based on deliberative Type 2 processing in these studies. These authors therefore employed the instructional manipulation paradigm to explore logical intuition in reasoning problems. In this paradigm, participants are required to indicate whether a conclusion is logically correct or not based on a logical instruction and judge believability based on a belief instruction. The rationale is that belief-based responses are fast and automatic, whereas logic-based responses are slow. When beliefs and logical responses conflict, this should only interfere with responses under the logical instruction condition and not those under the belief instruction condition. In fact, Handley et al. (2011) found that participants had lower accuracy and longer response times for conflict than no-conflict problems, irrespective of whether they were instructed to respond based on belief or logic. The processing of logic thus appears to be intuitive and rapid to the extent that it can influence rapid belief-based responses.

Drawing on Handley et al. (2011), Pennycook et al. (2014) employed the instructional manipulation paradigm to explore logical intuition in response to base-rate problems, asking participants to respond either under belief or statistics instructions. Their findings indicated that whether a problem was presented as a conflict affected participants’ performance in response to both statistics and belief instructions, reflected in probability estimates, response times, and confidence ratings. These results suggested that participants intuitively process base rates, allowing them to influence rapid belief-based responses. Thompson et al. (2018) replicated Pennycook et al.’s (2014) findings when exploring individual differences in logical intuition.

However, there are two major shortcomings with Pennycook et al.’s (2014) study. First, a traditional version of the base-rate task was used that included long text and was thus less sensitive to response time, which is the most direct and commonly used measure for assessing logical intuition. This may have contributed to these authors’ finding that the effect of logic was reflected in probability estimates and confidence ratings but not in response times. Second, and more importantly, that study only investigated base-rate problems with extreme base rates (e.g., 995/5), in which the contrast between heuristic intuition and probabilistic rules was apparent. One criticism of logical intuition studies for base-rate problems pertains to whether logical intuition exists exclusively in response to extreme base-rate problems. It remains to be verified whether the logical intuition effect also occurs in response to moderate base rates (e.g., 700/300) and moderate base rates with small absolute values (e.g., 70/30). Bago and De Neys (2020) and Yang et al. (2023a) explored logical intuition in response to moderate base rates, while Pennycook et al. (2014) and Yang et al. (2023b) considered moderate base rates with small absolute values. These studies yielded inconsistent conclusions and relied exclusively on the conflict detection paradigm. As mentioned above, that paradigm explicitly requires participants to respond deliberatively, which may confound the effect of logical intuition. Further investigation using the instructional manipulation paradigm is thus needed.

To address this issue, we explored logical intuition of base-rate problems using a rapid-response version of the instructional manipulation paradigm. We focused on logical intuition in response to extreme base rates in experiment 1. Experiment 2 considered logical intuition in response to moderate base rates, while experiment 3 examined logical intuition in response to moderate base rates with small absolute values. We predicted that participants would quickly and intuitively process base-rate information across the three experiments and that this processing would influence their belief-based and statistics-based judgments. As such, participants were expected to exhibit lower probability estimates, longer response times, and lower confidence ratings for conflict problems under both belief and statistics instructions.

## 2. Experiment 1

In the instructional manipulation paradigm, participants are asked to complete base-rate tasks based on either beliefs or statistics. Using this paradigm, we investigated the logical intuition of extreme base rates with the rapid-response version of the base-rate task. Belief instructions guided participants to ignore base-rate information and respond only using stereotypes according to their beliefs. In contrast, participants were asked to focus on the base rates of the problems and answer using statistical information, ignoring the stereotypical information under statistics instructions. We hypothesized that under both belief and statistical instructions, participants would show lower probability estimates, longer response times, and lower confidence ratings in answers for conflict problems than for no-conflict problems.

### 2.1. Method

#### Participants

G*Power 3.1 was employed to determine the necessary sample size with 80% power, an alpha level of 0.05, and a medium effect size (*f* = 0.25) based on the main effect (Faul et al., 2009). A minimum of 24 participants was required. Participants who signed up for the experiment were shown a classic base-rate problem and asked whether they had encountered the same or similar problems before. If they had, they would not have participated in the formal experiment. We recruited 45 participants (24 females, *M* = 19.84, *SD* = 1.41). After completing the experiment, participants were given 5 RMB as a token of appreciation.

### 2.2. Materials

Each participant responded to 28 base-rate problems, which comprised four practice problems and 24 test problems. Base-rate scenarios were translated and adapted from Pennycook et al. (2015). Before the experiment, we recruited 33 participants to rate the compatibility between the two groups and personality descriptions in each base-rate problem (e.g., how well IT engineers match with the trait “strong”). The scale ranged from 1 to 7, with higher numbers representing a closer match. For the 28 base-rate scenarios, 56 ratings were created, comprising 28 high-match and 28 low-match ratings. The high-match ratings (*M* = 6.02, *SE* = 0.13) were significantly higher than the low-match ratings (*M* = 3.46, *SE* = 0.16), *t*(32) = 10.58, *p* < 0.001, Cohen’s *d* = 1.84. This indicated that one of the two groups could trigger heuristic intuitions, suggesting the materials were suitable for constructing base-rate problems. Four of the 28 problems were randomly selected as practice problems, with the remaining 24 used as test problems. Participants involved in the compatibility ratings did not participate in the experiments.

In the experiment, participants were required to answer 12 no-conflict and 12 conflict items, created by randomly assigning scenarios to the respective categories. In no-conflict items, base rates and stereotypes aligned, whereas in conflict items, they did not (see Table 1). We used three extreme base rates in the experiment—995/5, 996/4, and 997/3—to reduce repetitiveness (Bago & De Neys, 2020; De Neys & Glumicic, 2008; Thompson et al., 2011). Each base-rate problem was randomly paired with one of these three base rates.

For all problems, there were four different combinations of each base-rate item, which differed by instruction type (i.e., belief or statistics instruction) and whether the target individual belonged to the larger or smaller group. Each combination included three problems for both no-conflict and conflict items. Table 1 shows the different combinations of no-conflict and conflict items for one scenario.

### 2.3. Procedure

E-Prime 3.0 software was employed to display the stimulus material and record the response times. Participants were presented with a rapid-response version of the base-rate task and were required to estimate the probability that an individual belonged to a particular group. Belief cues required participants to estimate the probability using their understanding of the world. We offered the following example to illustrate: “If a person on a city street is wearing shabby clothes and begging, then, based on real-world knowledge, there is a high probability that this person is homeless”. In contrast, statistics cues required participants to focus on the probability. In the above example, as only a small percentage of people in a city are homeless, the probability that the person is homeless would be low. Participants were also required to assess how confident they were in their probability judgments. The presentation order of the problems was randomized.

Each trial began with a 1000 ms fixation cross, followed by the instructional cue (e.g., “belief”) for 2000 ms. After this, a sentence describing sample information (e.g., “This study contains IT engineers and boxers”) was displayed for 2000 ms. Stereotype descriptions (e.g., “Participant A is strong”) then appeared for 2000 ms. The base-rate information (e.g., “There are 995 IT engineers and 5 boxers”) was then presented for 2000 ms. Next, the entire problem, including the question, appeared on the screen. At this point, the participants could click on the numbers to enter their probability estimate and, once entered, click “Done.” Finally, participants were asked to rate their confidence.

### 2.4. Results

#### 2.4.1. Scoring

Our method for probability estimation scoring was adapted from Pennycook et al. (2014). The data were recorded so that high scores always indicated correct responses in line with the instruction, which made interpretation of the data easier. Taking the base-rate scenarios presented in Table 1 as an example, for the statistics instructional cue, a lower probability estimate for Participant A being a boxer indicates compliance with the instruction, so the estimate is subtracted from 100, with higher scores indicating correct responses. For the probability of Participant A being an IT engineer, a higher estimate also indicates compliance. Under the belief instructional cue, a lower estimate for A being an IT engineer indicates compliance and requires subtraction from 100. For Participant A, being a boxer, a higher estimate is correct. Through recoding, higher probability estimates always represented compliance with the instructions and were considered correct.

#### 2.4.2. Missing Data

In 17 trials (1.57% of the total), participants entered a probability estimate greater than 100 or pressed “Done” without entering a probability estimate. These trials were deleted.

#### 2.4.3. Data Analysis

We conducted repeated-measures ANOVAs using SPSS 21.0 to examine the effects of conflict and instruction on probability estimates, response times, and response confidence.

#### 2.4.4. Probability Estimates

A 2 (conflict: conflict, no-conflict) × 2 (instruction: belief, statistics) repeated-measures ANOVA was conducted on probability estimates. Probability estimates for no-conflict problems (*M* = 82.70, *SE* = 1.97) were higher than those for conflict problems (*M* = 61.20, SE = 2.27), *F*(1, 44) = 64.32, *p* < 0.001, $\eta_{p}^{2}$ = 0.59. We set the parameters to an alpha level of 0.05, a sample size of *N* = 45, and an effect size of 1.20 (converted from $\eta_{p}^{2}$) for the post hoc power analysis using G*Power, showing that there was a sufficient power to detect this main effect (power = 1.00). The main effect of instruction was not significant, *F*(1, 44) = 3.04, *p* = 0.09, $\eta_{p}^{2}$ = 0.07. The interaction between conflict and instruction was not significant, *F*(1, 44) = 1.01, *p* = 0.32, $\eta_{p}^{2}$ = 0.02. Table 2 presents descriptive statistics.

#### 2.4.5. Response Times

Before conducting the ANOVA on the response times, we first performed a natural logarithm transformation. Subsequently, we carried out the Shapiro–Wilk test, revealing that the *p*-value was greater than 0.05. This indicates that the transformed response time data adhered to a normal distribution. Then, we ran a 2 (conflict: conflict, no-conflict) × 2 (instruction: belief, statistics) repeated-measures ANOVA on transformed response times. Transformed response times for no-conflict trials (M = 6145.89, SE = 350.11) were significantly faster than for conflict trials (M = 7560.43, SE = 507.75), *F*(1, 44) = 14.18, *p* < 0.001, $\eta_{p}^{2}$ = 0.24. The post hoc power analysis showed that there was sufficient power to detect this main effect (power = 1.00). The main effect of instruction was not significant, *F*(1, 44) = 1.94, *p* = 0.17, $\eta_{p}^{2}$ = 0.04, nor was the interaction between conflict and instruction, *F*(1, 44) = 1.32, *p* = 0.26, $\eta_{p}^{2}$ = 0.03 (Table 2 presents the original values of the response times).

#### 2.4.6. Response Confidence

We performed a 2 (conflict: conflict, no-conflict) × 2 (instruction: belief, statistics) repeated measures ANOVA on confidence ratings. We found a significant main effect of problem type, *F*(1, 44) = 11.75, *p* = 0.001, $\eta_{p}^{2}$ = 0.21. Participants’ confidence for no-conflict problems (*M* = 5.54, *SE* = 0.11) was significantly higher than for conflict problems (*M* = 5.27, *SE* = 0.13). The post hoc power analysis showed that there was sufficient power to detect this main effect (power = 1.00). No other significant effects were found (all *F*s < 1) (Table 2).

### 2.5. Discussion

In experiment 1, we explored the intuitive processing of extreme base rates using a rapid-response version of base-rate problems based on the instructional manipulation paradigm. We found that participants’ probability estimates for conflict problems were lower than those for no-conflict problems under both belief and statistics instruction conditions. Consistent results were obtained regarding response times and confidence ratings, with participants taking longer and having lower confidence ratings for conflict problems compared to no-conflict problems. Our findings suggest that participants intuitively processed base rates in a way that allowed them to influence rapid belief-based responses, indicating people have logical intuitions for base-rate tasks, at least when base rates are extreme. The results suggest that the non-significant response time findings reported in Pennycook et al.’s (2014) experiment 1 were likely caused by excessively long text. In addition, unlike Pennycook et al.’s (2014) experiment 1, we did not find lower response time under statistics instruction than belief instruction. This difference may be because participants in Pennycook et al. (2014) had to combine multiple pieces of descriptive information when answering based on beliefs. Additionally, the main effect of the conflict of probability estimates in this experiment ($\eta_{p}^{2}$ = 0.59) is greater than that of Pennycook et al.’s (2014) experiment 1 ($\eta_{p}^{2}$ = 0.48), which may be due to the fact that the effect of descriptive information was relatively weakened in this experiment. However, we did not find a similar pattern in confidence ratings, which may be influenced by additional factors, such as processing fluency (Thompson et al., 2013).

## 3. Experiment 2

Experiment 1 explored the logical intuition for base-rate problems with extreme base rates, where the contrast between the heuristic intuition and the base rate was prominent. One criticism of the study of logical intuition is its focus on elementary principles, such as extreme base rates. Changing the rate from extreme to moderate would reduce the contrast between heuristic intuition and base rates, with the strength of logical intuition thus becoming weaker (Bago & De Neys, 2020). The boundary condition of logical intuition—specifically, whether people possess logical intuitions in situations that cue weak logical intuition—has been a topic of debate in the field (De Neys & Pennycook, 2019). Pennycook et al. (2012) demonstrated that individuals exhibit logical intuition effects for extreme but not moderate base-rate problems (though see Yang et al., 2023a, 2023b). These studies were, however, based on the conflict detection paradigm and may not have captured purely logical intuitive effects.

In experiment 2, we used the instruction paradigm to explore the logical intuitions involved in moderate base-rate problems. Unlike experiment 1, which presented instructional cues first, the cues in this experiment were presented only once all parts of the problem had appeared, avoiding the processing of only specific information.

### 3.1. Method

#### 3.1.1. Participants

The necessary sample size, again calculated using G*Power 3.1 with parameters identical to experiment 1, was 24 participants. We recruited 41 participants (13 females, *M* = 19.29, *SD* = 1.54). All participants were unfamiliar with base-rate problems. After the experiment was completed, participants received 5 RMB as a token of appreciation.

#### 3.1.2. Materials

The experimental materials were consistent with those used in experiment 1 except for the extremity of the base rates. Three moderate base rates were used as follows: 700/300, 710/290, and 720/280. These values were selected to minimize repetitiveness (Yang et al., 2023a). For each base-rate problem, one of the three base rates was used randomly.

#### 3.1.3. Procedure

The procedure was identical to experiment 1 except for an adjustment to the presentation time of the instructional cues. These cues were only displayed once the entire base-rate problem was presented. Each item began with a fixation cross displayed for 1000 ms, followed by the sample information, stereotype description, and base-rate information, each shown for 2000 ms. Subsequently, the entire problem and the instructional cue, including the question, appeared on the screen. Participants entered their probability estimate and clicked “Done”. They then rated their confidence.

### 3.2. Results

#### 3.2.1. Missing Data

We discarded 13 invalid trials, which accounted for 1.32% of the total trials.

#### 3.2.2. Probability Estimates

We conducted a 2 (conflict: conflict, no-conflict) × 2 (instruction: belief, statistics) repeated-measures ANOVA on probability estimates. We found a main effect of conflict. Probability estimates for no-conflict items (*M* = 67.06, SE = 1.56) were higher than those for conflict items (*M* = 57.31, SE = 1.88), *F*(1, 40) = 22.00, *p* < 0.001, $\eta_{p}^{2}$ = 0.36. There was a significant main effect of instruction. Probability estimates based on belief instruction (*M* = 65.19, *SE* = 1.95) were higher than those based on statistics instruction (*M* = 59.67, *SE* = 1.30), *F*(1, 40) = 11.34, *p* = 0.02, $\eta_{p}^{2}$ = 0.22. The post hoc power analyses showed that there was sufficient power to detect the main effect of conflict and instruction (power = 1.00). The interaction between conflict and instruction was not significant, *F*(1, 40) < 1. Table 3 presents descriptive statistics.

#### 3.2.3. Response Times

We conducted a natural logarithm transformation on the response times and performed a Shapiro–Wilk test, which indicated that the *p*-value was greater than 0.05. A 2 (conflict: conflict, no-conflict) × 2 (instruction: belief, statistics) repeated measures ANOVA was conducted on transformed response time. There was a significant main effect of conflict. Participants took longer on conflict problems (M = 6681.47, SE = 410.99) than on no-conflict problems (M = 6090.37, SE = 346.24), *F*(1, 40) = 6.38, *p* = 0.02, $\eta_{p}^{2}$ = 0.14. The post hoc power analysis showed that there was sufficient power to detect this main effect (power = 1.00). The main effect of instruction was not significant, *F*(1, 40) = 1.44, *p* = 0.24, $\eta_{p}^{2}$ = 0.04, nor was the interaction between conflict and instruction, *F*(1, 40) = 1.30, *p* = 0.26, $\eta_{p}^{2}$ = 0.03 (see Table 3 for the original values of the response times).

#### 3.2.4. Response Confidence

A 2 (conflict: conflict, no-conflict) × 2 (instruction: belief, statistics) repeated measures ANOVA was performed on confidence ratings. There was a main effect of problem type. Participants’ confidence was higher on no-conflict problems (*M* = 5.66, *SE* = 0.11) than on conflict problems (*M* = 5.50, SE = 0.12), *F*(1, 40) = 5.76, *p* = 0.02, $\eta_{p}^{2}$ = 0.13. According to the post hoc power analysis, there was a sufficient power to detect this main effect (power = 1.00). The main effect of conflict was not significant, *F*(1, 40) < 1, nor was the interaction between conflict and instruction, *F*(1, 40) = 1.14, *p* = 0.29, $\eta_{p}^{2}$ = 0.03 (see Table 3).

### 3.3. Discussion

In experiment 2, we use the instructional manipulation paradigm to explore whether participants intuitively processed moderate base rates (e.g., 700/300). Instructional cues were presented at the end of each trial, once the entire problem had appeared, rather than at the beginning. This change was made to avoid the limitation of experiment 1, where participants may have focused only on information related to instructional cues. The results from experiment 2 indicated that participants’ probability estimates for conflict problems were lower than for no-conflict problems in both the belief and statistics instructional conditions. This suggests that conflict influenced both types of judgment. Data on response times and confidence ratings were consistent with probability estimates. Overall, this experiment showed that logical intuition is used in response to moderate base rates, expanding the boundary conditions for the logical intuition effect in base-rate problems. The results of experiment 2 were consistent with findings from Pennycook et al. (2015) and Yang et al. (2023a, 2023b), obtained using the conflict detection paradigm. In addition, this experiment found that the probability estimate under the belief instruction was larger than that under the statistical instruction ($\eta_{p}^{2}$ = 0.22), indicating a large effect size. This is different from experiment 1 of this study and the studies based on extreme base rates by Pennycook et al. (2014). This shows the impact of the extremity of base rates.

## 4. Experiment 3

In experiment 2, we investigated the use of logical intuition in response to moderate base-rate information, focusing on rates with large absolute values (e.g., 700/300). In experiment 3, we focused again on moderate base rates but this time with small absolute values (e.g., 70/30). Rates with large absolute numbers are generally perceived as more probable than identical rates presented with small absolute numbers (Denes-Raj & Epstein, 1994). For example, Bonner and Newell (2010) found that individuals tended to view 10/100 as more probable than 1/10. This shows that the scales of the absolute value may affect logical intuition.

Previous research on the logical intuition of moderate base rates with small absolute values has yielded different conclusions. Pennycook et al. (2012) used response times as the sole dependent variable to explore logical intuition in moderate base rates with small absolute values, finding no evidence of such intuition. In contrast, Yang et al. (2023b) used multiple measures to find logical sensitivity to moderate base rates with small absolute values. As mentioned earlier, however, the conflict detection paradigm may not be the most effective way to investigate logical intuition. Further investigation using the instructional manipulation paradigm is therefore necessary.

In addition, in experiments 1 and 2, we used a within-subjects instructional manipulation where participants switched from statistics-based instructions to belief-based instructions (or vice versa). Switching might lead participants to experience greater interference due to response conflict. Moreover, the switching itself may have confounded the experimental results. To rule out potential confounders, in experiment 3 we asked participants to first complete all items under the statistics-based instruction and then respond to the tasks under the belief-based instruction, or vice versa.

### 4.1. Method

#### Participants

A power analysis with identical parameters to experiments 1 and 2 showed a minimum of 24 participants was required. We recruited 45 participants (21 females, *M* = 18.91, *SD* = 1.08). All participants had not encountered base-rate problems before. Again, participants were given a 5 RMB prize after the experiment.

### 4.2. Materials

The scenarios for the base-rate problems were consistent with the two previous experiments. The base rates used were 70/30, 71/29, and 72/28. One of the three base rates was randomly applied to each problem (Yang et al., 2023b).

### 4.3. Procedure

Participants first answered all problems under the belief-based instructions and then completed all the items under the statistics-based instructions, or vice versa. The order in which participants answered the belief-based or statistics-based problems was random, and within each instruction condition, the presentation order of the items was also random. The procedure for a single trial was the same as in experiment 2.

### 4.4. Results

#### 4.4.1. Missing Data

We removed 18 invalid trials, representing 1.67% of the total trials.

#### 4.4.2. Probability Estimates

We conducted a 2 (conflict: conflict, no-conflict) × 2 (instruction: belief, statistics) repeated-measures ANOVA on probability estimates. There was a significant main effect of conflict, *F*(1, 44) = 36.21, *p* < 0.001, $\eta_{p}^{2}$ = 0.45. Participants’ probability estimates for no-conflict problems (*M* = 68.59, *SE* = 1.54) were higher than for conflict problems (*M* = 56.98, *SE* = 1.55). A significant effect of instruction was observed, *F*(1, 44) = 5.01, *p* = 0.03, $\eta_{p}^{2}$ = 0.10, with participants providing higher probability estimates for belief-based instruction trials (*M* = 65.82, *SE* = 2.16) than for statistics-based instruction trials (*M* = 59.76, *SE* = 1.39). According to the post hoc power analyses, there were sufficient powers to detect the main effect of conflict and instruction (powers = 1.00). The interaction between conflict and instruction was not significant, *F*(1, 44) < 1. Descriptive statistics are presented in Table 4.

#### 4.4.3. Response Times

A natural logarithm transformation was applied to the response times, and a Shapiro–Wilk test was conducted, showing a *p*-value higher than 0.05. A 2 (conflict: conflict, no-conflict) × 2 (instruction: belief, statistics) repeated-measures ANOVA was performed on transformed response times. The results revealed a main effect of conflict, *F*(1, 44) = 7.13, *p* = 0.01, $\eta_{p}^{2}$ = 0.14, with participants’ response times being significantly lower for no-conflict problems (M = 6152.01, SE = 347.91) than for conflict problems (M = 6546.84, SE = 350.96). The post hoc power analysis showed that there was sufficient power to detect this main effect (power = 1.00). The main effect of instruction was not significant, *F*(1, 44) < 1, nor was the interaction between conflict and instructions, *F*(1, 44) < 1 (Table 4 presents the original values of the response times).

#### 4.4.4. Response Confidence

We ran a 2 (conflict: conflict, no-conflict) × 2 (instruction: belief, statistics) repeated measures ANOVA on confidence ratings. The results revealed a significant main effect of conflict, *F*(1, 44) = 4.30, *p* < 0.05, $\eta_{p}^{2}$ = 0.09, indicating that participants reported higher confidence ratings for no-conflict problems (*M* = 5.82, *SE* = 0.13) than for conflict problems (*M* = 5.69, *SE* = 0.13). The post hoc power analysis showed that there was sufficient power to detect this main effect (power = 1.00). The main effect of instruction was not significant, *F*(1, 44) < 1. The interaction between conflict and instruction was also not significant, *F*(1, 44) < 1 (see Table 4).

### 4.5. Discussion

In experiment 3, we explored the logical intuition of moderate base rates with small absolute values (e.g., 70/30) with an instructional manipulation. We asked participants to respond to all belief-based tasks first, followed by statistics-based problems, or vice versa, to avoid confusion from switching between the two cues. Results showed that participants’ probability estimates were significantly lower for conflict problems than for no-conflict problems in both the belief-based and statistics-based instruction conditions, indicating that conflict influenced judgments in both contexts. Response times and confidence ratings aligned with the observed probability estimates, suggesting participants intuitively processed moderate base rates with small absolute values. We compared this experiment with experiment 3 in Pennycook et al. (2014), which also avoids the effect of switching. We found the main effect of instruction on probability estimates, with a medium effect size, suggesting the impact of extremity. This contrasts with experiment 3 in Pennycook et al. (2014), which did not find a main effect of instruction using extreme base rates.

The results of experiment 3 align with the findings of Yang et al. (2023b), which were based on the conflict detection paradigm, but are inconsistent with those of Pennycook et al. (2012). This discrepancy might be because Pennycook et al. (2012) used traditional base-rate problems and treated response times as the only dependent variable. Traditional base-rate problems have a lengthy text that affects the sensitivity of response times as a dependent variable (Ferreira et al., 2022).

### 4.6. General Discussion

The default–interventionist model suggests that base-rate neglect occurs because the processing of descriptive information relies on rapid Type 1 processing, whereas the processing of base-rate information relies on slow Type 2 processing (Evans & Stanovich, 2013). Pennycook et al. (2014) explored this hypothesis using the instructional manipulation paradigm and did not find a one-to-one correspondence between processing type and content. The present study built on this foundation by further investigating logical intuitions underlying base-rate problems using a rapid-response version of the base-rate task and the same instructional manipulation paradigm. Our rationale for using the instructional manipulation paradigm was that belief-based reasoning responses are typically fast and automatic, whereas statistics-based responses are typically slow and require more time. When beliefs and logic conflict, this conflict should primarily interfere with responses based on statistical instructions, but not those based on belief instructions.

In experiment 1, we tested logical intuition for extreme base rates. Instructions were presented before each trial began, which is one of the initial approaches based on the instruction paradigm used to investigate logical intuition in reasoning problems. In experiments 2 and 3, we examined logical intuition in response to moderate base rates and moderate base rates featuring small absolute values, respectively. Instructions were presented at the end of each trial to prevent participants from focusing solely on the information related to the instructions. By requiring participants to finish all of the problems for one type of instruction before going on to the problems for the other, experiment 3 minimized the possible impact of instruction switching. The results of the three experiments indicated that a belief–statistics conflict impacted participants’ performance under both the statistics instruction and the belief instruction. This was evidenced by a decrease in probability estimates, increased response time, and reduced confidence in estimates. A plausible explanation for this pattern of findings is that participants intuitively processed base rates, which allowed the belief–statistics conflict to influence their rapid belief-based responses, thereby highlighting the effect of logical intuition in base-rate problems.

Our study extends Pennycook et al.’s (2014) study, which investigated intuitive logic in response to base-rate problems using the instructional manipulation paradigm. Pennycook et al. (2014) used traditional base-rate problems that involved lengthy text, resulting in a non-significant effect on response-time data. The present study used the rapid-response version of the base-rate task, which minimized confusion by reducing the time participants spent reading each problem. Our purer response time index indeed showed an effect of intuitive processing of base rates.

More importantly, while Pennycook et al. (2014) focused exclusively on logical intuition for extreme base-rate problems, the present study explored logical intuition in response to both moderate base-rate problems and those having moderate base-rates with small absolute values. A key question is whether logical intuition arises solely in response to extreme base-rate problems, where the contrast between heuristic intuition and the base-rate is clear, or whether it also occurs in response to moderate base-rate problems where this contrast is less obvious. This question pertains to the boundary conditions of logical intuition, and our study contributes to this discussion. The data from experiments 2 and 3 showed that participants showed logical intuition for both moderate base rates and moderate base rates with small absolute values. This finding extends the boundary conditions for logical intuition in base-rate problems. In addition to manipulating the extremes of base rates, future research could explore more realistic scenarios from everyday life to investigate boundary conditions using the instructional manipulation paradigm. For example, Pennycook et al. (2012) developed implicit base-rate problems in which the base-rate was not explicitly provided, but compatibility ratings indicated that one of the two groups consistently had a higher base-rate than the other.

Yang et al. (2023a) used the conflict detection paradigm to examine the boundary conditions of logical intuition in the base-rate problem, and their results are consistent with those reported here. However, as discussed, evidence derived from conflict detection may stem from deliberate processing, as participants in conflict detection studies are explicitly asked to engage in deliberative thinking (see also Pennycook et al., 2015; Yang et al., 2023b). The instructional manipulation paradigm of this study overcomes this shortcoming, making experimental results more reliable.

The current research has important theoretical implications for dual-process theories. Our results do not align with the default–interventionist model of dual-process theories, which suggests that access to logical structure depends on Type 2 processing. The findings of this study suggest that logical structure can also be accessed through Type 1 processing, supporting the logical intuition view. This perspective corresponds with more recent iterations of dual-process models, including the parallel processing model (Handley & Trippas, 2015), the hybrid model (De Neys, 2012), and the three-stage model (Pennycook et al., 2015). All of these models incorporate logical intuition into their frameworks. The parallel processing model suggests that the processing of beliefs is activated in parallel with the processing of logical rules and that both rely on Type 1 and Type 2 processing. The final response is influenced by task demands, available working memory resources, and time pressure. The hybrid and three-stage models posit that Type 1 processing produces different intuitions at the beginning of the process, with logical intuition being one of them. The relative strength of logical intuition in relation to other intuitions (e.g., heuristic intuitions) determines the final response (Bago & De Neys, 2020; Pennycook et al., 2015). In addition, De Neys (2023) developed a more viable general model of dual-process theories that aims to explain a wider range of human behavior using the dual-process framework. This model suggests that dual-process theory contains four components: intuitive activation, uncertainty monitoring, deliberation, and feedback. The model posits that during the intuitive activation stage, Type 1 processing produces various intuitions, including logical intuitions. This view is similar to the hybrid and three-stage models. It should be noted that the primary objective of this study was to explore logical intuition effects in reasoning problems, not to distinguish between different types of dual-process theory models.

From a general perspective, the existence of logical intuition has important implications for discussing human rationality. Over the past decade, the low accuracy rates of educated adults in classical reasoning tasks have led researchers to question the rationality of humans (Kahneman, 2011). The default–interventionist model suggests that people follow heuristic intuitions blindly with no consideration of logic or probability, encouraging a rather pessimistic view of human rationality (Frederick, 2005). Our findings support a different view, suggesting that people do have intuitive logic. Specifically, our findings suggest that people do not ignore base rates when confronted with base-rate problems. This was evident in how the presence or absence of conflict in a problem influenced participants’ performance when they were asked to make belief-based judgments that did not require deliberative thinking. This effect persisted even when base rates were not extreme. Our study therefore paints a more optimistic picture of human rationality.

In the current research, we used response-based indicators as dependent variables. Future research could combine an instructional manipulation paradigm with physiological methods to further explore logical intuition in base-rate problems. An advantage of this approach is that it offers the potential to investigate whether the effects of logical intuition are present before participants respond, which would provide more solid support for the existence of logical intuition. For example, Purcell et al. (2023) investigated logical intuition effects in reasoning problems using the instructional manipulation paradigm combined with eye movement measures. The results showed that both response-independent pupil dilation and gaze reflected the effects of logical intuition, which existed before participants responded. Other physiological indices, including EEG and fMRI, could also be used (Bago et al., 2018; De Neys et al., 2008). Additionally, future research could also use strict time pressure and cognitive load to further limit Type 2 processing when using the instructional manipulation paradigm. This would help obtain more pure, intuitive responses (Bago & De Neys, 2017; Raoelison et al., 2021).

## 5. Conclusions

This study found that participants can process extreme base rates intuitively. The findings expand the boundary conditions for logical intuition by confirming that participants have logical intuition in response to moderate base rates, including those with small absolute values. Our findings contradict the default–interventionist model, instead favoring dual-process theories that take logical intuition into account. We thus provide more evidence for the existence of human rationality.

## Figures and Tables

**Table 1:** Examples of problems with different combinations of instruction type and group.

Conflict	No-Conflict
Larger group	Larger group
This study includes IT engineers and boxers.	This study includes IT engineers and boxers.
Participant A is strong.	Participant A is strong.
There are 995 IT engineers and 5 boxers.	There are 995 boxers and 5 IT engineers.
What is the probability that A is an IT engineer?	What is the probability that A is a boxer?
Statistics: High	Statistics: High
Belief: Low	Belief: High
Smaller group	Smaller group
This study includes IT engineers and boxers.	This study includes IT engineers and boxers.
Participant A is strong.	Participant A is strong.
There are 995 IT engineers and 5 boxers.	There are 995 boxers and 5 IT engineers.
What is the probability that A is a boxer?	What is the probability that A is an IT engineer?
Statistics: Low	Statistics: Low
Belief: High	Belief: Low

**Table 2:** Probability estimates, response times, and confidence under belief and statistics instructions for each problem type in experiment 1 (standard errors in parentheses) (N = 45).

Variable	Belief Instruction		Statistics Instruction	
	Conflict	No Conflict	Conflict	No Conflict
Probability estimates (%)	64.96 (3.20)	83.62 (2.24)	57.45 (3.63)	81.76 (2.17)
Response time (ms)	7254.93 (692.27)	6060.29 (352.92)	7865.88 (512.12)	6231.48 (431.30)
Confidence	5.30 (0.14)	5.61 (0.12)	5.31 (0.15)	5.53 (0.13)

**Table 3:** Probability estimates, response times, and confidence under belief and statistics instructions for each problem type in experiment 2 (standard errors in parentheses) (N = 41).

Variable	Belief Instruction		Statistics Instruction	
	Conflict	No Conflict	Conflict	No Conflict
Probability estimates (%)	60.67 (2.49)	69.72 (2.16)	53.96 (2.49)	64.39 (1.33)
Response time (ms)	6722.25 (457.54)	6390.20 (419.85)	6640.70 (456.24)	5790.55 (362.10)
Confidence	5.43 (0.14)	5.66 (0.12)	5.57 (0.14)	5.66 (0.13)

**Table 4:** Probability estimates, response times, and confidence under belief and statistics instructions for each problem type in experiment 3 (standard errors in parentheses) (N = 45).

Variable	Belief Instruction		Statistics Instruction	
	Conflict	No Conflict	Conflict	No Conflict
Probability estimates (%)	61.13 (2.80)	70.51 (1.91)	52.83 (2.72)	66.68 (1.92)
Response time (ms)	6679.42 (487.14)	6283.44 (541.17)	6414.26 (374.30)	6020.58 (319.27)
Confidence	5.70 (0.13)	5.81 (0.13)	5.68 (0.15)	5.82 (0.13)

## Data Availability

The original data presented in the study are openly available in FigShare at the following link: https://doi.org/10.6084/m9.figshare.28060547.v1.
